# Assessment of the Relationship between Fear of Cancer Recurrence, Spiritual Well-Being, and Mental Health among Cancer Patients: A Cross-Sectional Study

**DOI:** 10.3390/nursrep14010024

**Published:** 2024-01-29

**Authors:** Agoritsa Londoudi, Konstantinos Skampardonis, Victoria Alikari, Paraskevi-Maria Prapa, Aikaterini Toska, Maria Saridi, Maria Lavdaniti, Sofia Zyga, Evangelos C. Fradelos

**Affiliations:** 1 General Hospital of Volos, 38 222 Volos, Greece; alontoudi@uth.gr; 2 Laboratory of Clinical Nursing, Department of Nursing, University of Thessaly, 41 500 Larissa, Greece; kskampard@uth.gr (K.S.); msaridi@uth.gr (M.S.); 3 Department of Nursing, University of West Attica, 122 43 Athens, Greece; vicalikari@uniwa.gr; 4 Department of Nursing, International Hellenic University, 570 01 Thessaloniki, Greece; maria_lavdaniti@yahoo.gr; 5 Department of Nursing, School of Health, University of the Peloponnese, 221 00 Tripoli, Greece; zygas@uop.gr

**Keywords:** cancer, fear of cancer recurrence, mental health, spirituality, insomnia

## Abstract

The purpose of this study was to examine the relationship between fear of cancer recurrence, spiritual well-being, and mental health among cancer patients. The study involved 217 patients undergoing chemotherapy. Data were collected with the following instruments: a demographic and clinical information recording form, the fear of cancer recurrence inventory, the Athens insomnia scale, the FACIT-Sp-12 scale for the assessment of spirituality, and the HADS scale for the assessment of mental health. For statistical analysis, SPSS statistical software was used, with the significance threshold set at 0.05;andtl number, *t*-test, one-way ANOVA, and multiple regression tests were used. The sample consisted of 217 cancer patients with a mean age of 63.7 years (SD = 11.6 years), 39.2% male and 60.8% female. The minimum value on the scale of fear of cancer recurrence was 0 and the maximum was 33 points, with a mean value of 14.1 points (SD = 8.2 points). The hospital scale of anxiety and depression was correlated, both in the dimension of anxiety and in the dimension of depression, significantly and positively with the scale of fear of cancer recurrence. Thus, greater fear of recurrence was associated with greater anxiety and depression. On the contrary, the correlations of anxiety and depression with the dimensions and the overall chronic disease treatment rating scale were significant and negative. So, greater spiritual well-being, in each domain and overall, were associated with less anxiety and depression. Finally, less fear of cancer recurrence was associated with finding greater meaning in life, greater peace, and overall greater spiritual well-being. In summary, fear of cancer recurrence is a predictor of psychological distress in cancer patients. However, spirituality can prevent the development of mental illness and FCR.

## 1. Introduction

Cancer patients face a trajectory marked by mental and physical pain that is connected to both diagnosis and treatment. One of the typical unmet demands mentioned by patients with both localized and metastatic cancer is a fear of recurrence or advancement of the disease [1]. According to a definition given by the American Cancer Society, fear of cancer recurrence (FCR) or progression is “fear, worry, or concern associated with the possibility that the cancer will return or progress” [2,3]. Fear of a cancer relapse has been linked to a diminished quality of life and psychosocial adjustment, greater mental distress, and a variety of physical symptoms, despite being frequently ignored by medical teams [4,5,6,7]. The phrase “a fear or worry that cancer will return or develop in the same or a different part of the body” is frequently used to describe the FCR [2].

It is widely accepted that FCR is recognized as the most common psychological burden experienced by cancer patients and survivors. According to previous studies, 39–97% of cancer survivors experience a distressing experience of cancer recurrence, and the range of manifestation of fear that this will occur varies from fear as a natural response to behavioral disturbances, depression, and anxiety [8]. In addition, the FCR is associated with a poor quality of life, functional impairment, and elevated healthcare expenses [9]. Furthermore, many studies conducted on survivors of various types of cancer, such as breast, colon, and cervical cancers, showed that the FCR was significantly associated with demographic, medical, and psychological characteristics, such as an early onset, a recent cancer diagnosis, a more advanced stage of cancer, a low level of satisfaction with care, limited social support, illness perception, experiencing intense pain, and disability. A study conducted in Korea reported associations between clinical-level FCR and communication, care coordination, functional social support, HRQoL, fatigue, sleep problems, and psychological distress when adjusted for age, gender, stage of cancer, co-morbidities, and time since cancer diagnosis. FCR was inversely related to social support and was positively correlated with symptom scales such as fatigue, pain, sleep problems, anxiety, and depression [10]. Similarly, a study of breast cancer survivors found that women with high morale and psychological flexibility had a lower FCR. In this study, we discussed the benefits of spirituality and religion for cancer patients as well as the interaction and relationship between these factors and the occurrence of a FCR, and proposed a meditation model [11].

Various studies reported that is crucial to examine the levels of the FCR in the early stages of therapy. According to Hong, Shin, and Jung, FCR is a mediating factor and can affect psychological distress, illness representation, and psychological adjustment to illness among patients who are undergoing chemotherapy [12]. A qualitative study conducted in Iran found that cancer patients were more worried about their lives due to poor recovery and a FCR. Anxiety in cancer survivors sometimes stems from concern about pathological patterns and possible additional therapy, which can lead to a fear of incomplete treatment. Concerns about cancer recurrence are based on physical symptoms and are closely related to follow-up time and medical examinations. Researchers report that cancer survivors have a high FCR requiring immediate attention and psychological support. This study highlights the importance of identifying the FCR in prostate cancer survivors and treating it with cognitive behavioral therapy [13]. The FCR model proposed by Simonelli and colleagues is a general conceptual framework for recognizing people and encounters through a FCR. According to the model, the unique experience of illness is produced by internal (e.g., physical symptoms, treatment side effects, psychological problems) and external (e.g., communication, clinical development) stimuli that initiate related psychological responses. With regard to the FCR, these symptoms are interpreted by mental processes, and physical symptoms are considered to be possible signs of relapse, especially when emotional pain is involved. Patients develop the diagnosis of their disease through self-assessment, resulting in different levels of FCR. The severity of FCR depends on the subjective assessment of the severity of the disease and the assessment of the risk that leads to an elevated FCR. This intense fear can lead to negative psychological effects such as panic attacks or depression. Importantly, this model demonstrates the diversity of factors in the development of a FCR by recognizing that contextual factors, including social context, personality, illness, and age, influence this assessment process [14].

The complex relationship between the FCR and insomnia is an important predictor for individuals navigating life after cancer treatment. Constant anxiety about the risk of cancer recurrence has been linked to the development of insomnia, a condition that disrupts sleep patterns, leading to a relationship of mutual influence.

Worrying about the possibility of cancer recurrence can lead to more anxiety and ultimately interfere with a person’s ability to achieve restful sleep. On the other hand, altering sleep habits can increase the psychological distress associated with cancer recurrence. A study that investigated the relationship between social constraints and insomnia among African American breast cancer survivors, with a focus on the mediating role of the FCR, found that the direct effect highlights that experiencing social constraints from family and friends significantly contributes to insomnia among African American breast cancer survivors. Additionally, the study revealed an indirect effect, demonstrating that the impact of social constraints on insomnia is partially mediated by the FCR. The findings suggest that negative social interactions may give rise to cognitive processes, such as the FCR, which in turn, affect sleep patterns. The researchers recommended family-based models of care that prioritize emotional support for survivors and their families as a potential strategy to address social constraints and mitigate their impact on sleep among African American breast cancer survivors [15].

With regard to a study conducted by Savard and Ivers among cancer patients undergoing chemotherapy, the researchers indicated that the FCR is a highly prevalent and persistent condition among cancer patients and increased efforts should be devoted to developing effective treatments; in addition, early interventions must be applied in those patients to prevent the problem from becoming chronic [16]. However, the findings in terms of the prevalence and the associated factors are sometimes contradictory in different studies. For example, according to a cross-sectional study among pancreatic cancer survivors and a systematic review, the reported results are conflicting and always support the association of the FCR with health-related parameters [17,18]. In addition, to our best knowledge, the FCR has not been well researched among Greek cancer patients. 

Thus, this study aims to examine the relationship between the fear of cancer recurrence and mental health, insomnia, and spirituality among cancer patients receiving or who have recently completed chemotherapy. 

## 2. Materials and Methods

A cross-sectional study was conducted among cancer patients who were receiving chemotherapy from a day clinic of the State Hospital of Volos, Greece. Convenience sampling was used to recruit participants. Inclusion criteria were patients older than 18 years, currently undergoing chemotherapy, and able to speak, read, and understand Greek. Patients previously diagnosed with psychiatric disorders or mental retardation were excluded. A total of 305 patients who attended the day clinic were assessed for eligibility. A total of 270 patients met the criteria to participate in the study and 236 agreed to participate (15.5% refused to participate). Finally, 217 patients provided full data and were included in the study.

### 2.1. Data Collection

#### Instrument

A five-part questionnaire was used for data collection.

1. The first part contained questions regarding socio-demographic and clinical information that were specifically designed for this study.
2. The FCRI-SF (short form of the fear of cancer recurrence inventory) is a measure of anxiety. It is frequently used to detect possible cases of clinically significant FCR. The FCRI has been widely utilized in observational and interventional investigations. The short version has 9 questions, in contrast to the full version, which has 42 questions [19]. The scale was translated in Greece for this study. Information regarding the validity of the Greek version of the scale is presented in the Results Section.
3. The functional assessment of the chronic illness therapy spiritual well-being scale (FACIT-Sp-12) questionnaire was developed in 1990 as a brief measure that assesses spirituality [20]. It is a self-administered questionnaire containing a twelve-item scale, four-point closed questions (0 = not at all, 1 = a little, 2 = somewhat, 3 = quite a bit, 4 = a lot), and three sub-domains of spiritual well-being, peace, meaning, and faith. It is an instrument that has been used in many studies and has been translated and validated in many languages. In the present study, the Greek version of the questionnaire weighted by Fradelos et al. 2016 [21] was used.
4. The AIS (Athens insomnia scale), based on the international classification of diseases, 10th edition (ICD-10) diagnostic criteria, are used to evaluate the severity of insomnia on the scale. The eight-question survey evaluates sleep onset, sleep length, sleep quality, frequency, duration of complaints, distress brought on by the experience of insomnia, and impairment with everyday functioning. Only the first five questions are included in a condensed version of the survey [22].
5. Zigmond and Snaith created the hospital anxiety and depression scale (HADS) in 1983 [23]. It is intended to give doctors a practical instrument for recognizing and measuring sadness and anxiety that is acceptable, trustworthy, valid, and simple to use. The scale’s function is dimensional rather than categorical. It is best used to identify individuals in a general hospital who require additional mental screening and assistance rather than to diagnose psychiatric diseases. The scale utilized in this investigation was the Greek version, as weighted by Michopoulos et al., 2008 [24].

### 2.2. Statistical Analysis 

The Kolmogorov–Smirnov test was used for testing the normality of distribution. For those values that were normally distributed, mean values and standard deviations (SDs) were used for their description, while for those that were not normally distributed, medians and interquartile ranges were also used. Absolute (N) and relative (%) frequencies were used for the description of qualitative variables. The Cronbach’s α index was used to test the reliability of the resulting factor measurements. To examine the link between two quantitative variables, the Spearman’s correlation coefficient was utilized. The comparison of quantitative variables between the two groups was carried out using the non-parametric Mann–Whitney U test. To compare numerical variables between more than two groups, the non-parametric Kruskal–Wallis test was employed. The Bonferroni adjustment, with a significance threshold of 0.05/n (n = number of comparisons), was employed to check for type I errors caused by multiple comparisons. The identification of independent factors required the use of linear regression analysis. The levels of significance were two-sided and statistical significance was set at 0.05. The statistical programs SPSS 26.0 and JASP 0.14.1.0 were used for the analyses.

### 2.3. Ethical Issues

Permission for the study was obtained from the Institutional Review Board (IRB) of the General Hospital of Volos and the study was carried out in accordance with the Declaration of Helsinki. An informed consent form and information regarding the option to withdraw from the study at any time was provided to all participants. To obtain informed consent, a member of the research team approached each participant and, after an extensive interview explaining the purpose and procedures of the study, each participant signed a consent form and completed a questionnaire.

## 3. Results

The sample consisted of 217 cancer patients with a mean age of 63.7 years (SD = 11.6 years). Participant demographics are given in Table 1.

Fear of cancer recurrence inventory was translated into Greek following the forward–backward translation method. The produced Greek version of the scale was administered to a small sample of 10 cancer patients to be tested for clarity of the content. All patients stated that the scale was appropriate and easy to understand. Then, the scale was distributed to 30 cancer patients twice within three weeks to test the stability of the scale during the first administration. The mean score was found to be 13.9 (7.9), while during the second scale administration, it was found to be 14 (7.8). A significant correlation was revealed between the occasions that the scale was administered, with r = 0.589 and *p* < 0.001, indicating the good degree of stability of the scale. Those data were not included in the final sample. Finally, the validity of the scale was examined in the total sample (n = 217) using the confirmatory factor analysis techniques. The results indicated that the one-factor solution of the Greek version of the fear of cancer recurrence inventory was acceptable as fit indications were found to be fair. More specifically, TLI was 0.808 (>0.8 and <0.9); CFI was 0.867, close to 0.9; and RMSEA was 0.076 and lower than 0.10. In general, the CFA verified the one-factor structure of the FCRI. 

Regarding the descriptive statistics of the fear of cancer recurrence inventory, a higher score implies a greater fear. The minimum value on this scale was 0 and the maximum was 33 points, with a mean value of 14.1 points (SD = 8.2 points). Taking into consideration the cut-off value of 13 for the scale, 102 patients (47%) experienced clinical levels of fear of cancer recurrence. Regarding spirituality, the scores in the subscales range from 0 to 16 points and the total ranged from 0 to 48. A higher score implies greater spiritual well-being. In this sample, the minimum value in the “meaning” dimension was 3 and the maximum was 16 points, with an average value of 13.6 points (SD = 2.6 units). The minimum value in the “peace” dimension was 0 and the maximum was 16 points, with a mean value of 10.7 points (SD = 3.2 points). The minimum value in the “faith” dimension was 1 and the maximum was 16 points, with a mean value of 12 points (SD = 3.6 points). The minimum value on the total scale was 9 and the maximum was 48 points, with a mean value of 36.3 points (SD = 7.7 points). According to the results of the Athens insomnia scale, the mean score was found to be 4.6 (4) and according to the cut-off point of the scale, 77 patients (35.5%) were suffering from insomnia. In terms of depression and anxiety in this sample, the minimum value in the anxiety dimension was 0 and the maximum was 21 points, with a mean value of 4.7 points (SD = 4.9 points). In this sample, the minimum value in the depression dimension was 0 and the maximum was 21 units, with a mean value of 4.4 units (SD = 4.7 units). A higher score implies greater anxiety or depression. The Cronbach’s α reliability coefficient was greater than 0.7 on all instruments, indicating acceptable reliability (Table 2). 

According to the bivariate analysis, occupation was found to have a statistically significant effect on the fear of cancer recurrence; more specifically, patients who stated household as their current occupation status reported higher levels of fear of cancer recurrence compared to other occupation statuses (F(5,211) = 3.029, *p* = 0.012). In addition, patients receiving intravenous therapy also reported higher levels of fear of cancer recurrence compared to those receiving oral or intramuscular therapy (F(3,213) = 7.728, *p* < 0.001). 

The Pearson correlation coefficient revealed statistically significant correlations between the fear of cancer recurrence and spiritualty, insomnia, and mental health; more specifically, positive correlations were observed the between fear of cancer recurrence, insomnia, depression, and anxiety, and negative correlations were observed between the fear of cancer recurrence and spirituality. Detailed results are presented in Table 3. 

According to multiple regression results, the fear of cancer recurrence can significantly increase depression, anxiety, and insomnia. Detailed results are presented in Table 4. 

The multiple regression results with the fear of cancer recurrence and spirituality dimensions as independent variables, and depression, anxiety, and insomnia as dependent variables, adjusted for demographics and other sample characteristics, are shown in Table 5. Meaning was the only independent predicting factor for the fear of cancer recurrence, insomnia, and anxiety, while all subscales of spirituality could predict lower scores of depression. Detailed results are presented in Table 5. 

## 4. Discussion

This study aimed to examine the FCR among cancer patients receiving chemotherapy. The prevalence of the FCR was found to be 49.8%. In addition, a strong correlation was observed between mental health, insomnia, and spiritual well-being with the presence or absence of fear of cancer recurrence (FCR). A greater FCR was associated with greater anxiety and depression. In contrast, in the hospital anxiety and depression scale (HADS), the correlations of anxiety and depression with the dimensions and the overall spirituality scale were significant and negative. Finally, less fear of cancer recurrence was associated with finding greater meaning in life, greater peace, and an overall greater spiritual well-being.

According to our results, almost half of the patients were experiencing a FCR. This finding agrees with previous studies indicating that cancer patients often experience a FCR. A recent systematic review of 87 studies from 13 countries included 9.311 respondents in the main analyses. On the FCRI-SF (range 0–36), 58.8% of respondents scored ≥13, indicating that a FCR was found across cancer types and continents and for all periods since cancer diagnosis [25]. According to the results of the bivariate analysis, only occupation and the type of administration of treatment were found to correlate with FCR. Although there are several factors correlated with FCR such as depression, anxiety, and health beliefs, when it comes to demographic and clinical information, the results are conflicting. According to Crist and Grunfeld [26], based on their literature review, the type of treatment is moderately associated with an increased FCR. Similarly, Shin et al. [10] reported that no clinical or sociodemographic factors were associated with the increased level of FCR. Another study in China reported that cancer-related and socio-demographic characteristics such as no religious beliefs, a lower family income, and treatment modality were found to affect the levels of FCR [27]. The heterogeneity of diagnosis (cancer type and stage), treatments, and cultural differences, along with the variety of sociodemographic characteristics, can partially explain the conflicting results, yet create a gap that future studies on the field of FCR can fill. 

In the present study, positive significant correlations were observed among the FCR, depression, anxiety, and insomnia. This result is in agreement with those of various studies in the field indicating that the FCR is associated with various psychological variables and can increase the psychological distress and burden of the patients. According to a recent cross-sectional study in Australia among breast cancer survivors, the FCR had a positive correlation with anxiety, depression, stress, and fatigue among the participants [28]. Those researchers indicated that the FCR can act as a mediating factor in transitioning from fatigue to psychological distress [27]. Similar results were also reported by Quan et al. [29]. According to their research, the fear of recurrence, invasive rumination, catastrophizing, and depression among cancer patients were significantly positively correlated, and the level of fear of recurrence was a significant positive predictor of the level of depression. Although they suggested different mechanisms and mediating variables, they also highlighted the relationship of the FCR and depression. It is known that patients suffering from anxiety or depression around cancer often think about the risk of disease recurrence [30]. A previous stud exploring this relationship [31] has shown that FCR, anxiety, and depression can coexist and intensify effects during treatment and recovery. Specifically, negative rumination was found to interact with the symptoms of the FCR, depression, and anxiety. In addition, studies have shown that psychological factors such as depression, anxiety, and stress play a greater role in the FCR than clinical factors such as mastectomy [32]. The relationship between depression and the FCR can be explained by the Lee-Jones FCR theory model, which states that the FCR has similar symptoms to anxiety and depression [33].

In addition, various studies also support the effect that the FCR can have on sleep and sleep quality among cancer survivors and cancer patients. Researchers have again suggested various models demonstrating that FCR can directly affect sleep quality among those individuals or that it requires mediating factors for this outcome to be observed [15,34]. In an observational study of breast cancer survivors, FCR was related to sleep problems, including reduced sleep duration and quality for patients and partners [35].

In our study, spirituality was found to be negatively associated with FCR, anxiety, depression, and insomnia. This result adds value to the general notion that suggests a positive relationship between spirituality and positive health outcomes, like fewer symptoms of anxiety and depression. Numerous studies are reporting that spiritual well-being is positively related to physical and mental health [36]. The significant positive effect that spirituality has on anxiety, depression, fear of recurrence, and insomnia is also reflected in our study, as well as in other studies that have been carried out from time to time. However, in all these studies, clarification and separation are made between spirituality and religiosity. Increased spirituality has been associated with better adjustment to adverse life events and effective coping [37]. According to a randomized controlled trial conducted by Carlson et al. [38], in which a spiritually oriented intervention was tested, found that meditation was more effective and reduced the levels of anger, anxiety, and tension compared to non-spiritual relaxation techniques. Moreover, according to our results, all subscales, including faith in the FACIT-Sp-12, affect the levels of depression. In general, according to the existing literature, there is a stronger relationship between spirituality and psychological health, compared to religiosity and psychological health [39,40]. A study conducted by Nelson et al. [39] examined the possible relationship between spirituality, religiosity, and depression among prostate cancer patients. The researchers hypothesized that spirituality can be a mediating factor between religiosity and depressive symptoms. They concluded that strong religious beliefs were only helpful in reducing depressive symptoms among those who found meaning in their religion. Both spirituality and psychological resilience have been found to play an important role in the efforts to cope with adverse life events.

Although the study indicated some interesting significant relationships between the variables under investigation, some limitations exist. First, the cross-sectional study design does not provide a deeper understanding of the FRC and its effect. Secondly, the sample is derived from a single facility and does not allow generalization of the results. Finally, the fact that there was no preregistration of this study can be considered as an additional limitation. 

Future studies must be conducted including variables such as fatigue, nausea, and other somatic symptoms, as well as variables such as social support, to better understand the relationship between the fear of cancer recurrence and contextual, social, and psychological factors. 

### Implications for Clinical Practice

Research on the FCR has important clinical implications. Periodic screening for the FCR is important to identify patients at risk, considering variables such as social restrictions and insomnia. To address the emotional needs of cancer survivors and their families, clinical interventions should combine family therapy with psychoeducation and counseling. Personal treatments, such as cognitive behavioral therapy, can be used for individual experiences. Collaboration between mental health professionals and oncologists is essential to develop comprehensive strategies for the management of the FCR. The ongoing nature of the FCR requires long-term maintenance, including regular review and the modification of support strategies.

## 5. Conclusions

According to our study, the fear of cancer recurrence acts on many levels and in combination with other factors, affecting the mental, physical, and spiritual health and well-being of patients. Studies such as this one, as well as others cited here, aim to raise awareness of these issues and result in the improved training of health professionals, nursing staff, and all kinds of caregivers, in providing more integrated care and adapting (on a case-by-case basis) care to their patients.

## Figures and Tables

**Table 1:** Sociodemographic and clinical information of the sample (n = 217).

		N	%
**Gender**	Male	85	39.2
	Female	132	60.8
Age (mean (SD); median (range))		63.7 (11.6)	66.0 (55.0–73.0)
Family status	Single	16	7.4
	Married	145	66.8
	Widowed	33	15.2
	Divorced	23	10.6
Number of children	0	22	10.1
	1	40	18.4
	2	120	55.3
	3	27	12.4
	>4	8	3.7
Place of residence	Urban	149	68.7
	Semi-urban	32	14.7
	Rural	36	16.6
Educational level	Elementary	73	33.6
	High school	91	41.9
	University	40	18.4
	Postgraduate	11	5.1
	None	2	0.9
Occupation	Public sector	25	11.5
	Private sector	18	8.3
	Freelancer	22	10.1
	Unemployed	17	7.8
	Pension	97	44.7
	Household	38	17.5
Type of cancer	Breast	81	37.3
	Lung	44	20.3
	Colon	34	15.7
	Ovarian	16	7.4
	Pancreatic	40	18.4
	Other	2	0.9

**Table 2:** Descriptive statistics for fear of cancer recurrence, spirituality, Athens insomnia scale, and hospital anxiety and depression scale.

	Min	Max	Mean (SD)	Median (Range)	Cronbach’s a
Fear of cancer recurrence inventory (FCRI)	0.0	33.0	14.1 (8.2)	13 (7–21)	0.88
Meaning	3.0	16.0	13.6 (2.6)	14 (12–16)	0.75
Peace	0.0	16.0	10.7 (3.2)	12 (9–13)	0.71
Faith	1.0	16.0	12 (3.6)	12 (10–15)	0.85
Total score on spirituality scale.	9.0	48.0	36.3 (7.7)	37 (33–42)	0.86
Athens insomnia scale	0.0	21.0	4.6 (4)	21	0.91
HADS—anxiety	0.0	21.0	4.7 (4.9)	3 (0–7)	0.91
HADS—depression	0.0	21.0	4.4 (4.7)	2 (0–7)	0.89

**Table 3:** Pearson correlation coefficients between fear of cancer recurrence and insomnia, spirituality, and mental health scales.

		HADS— Anxiety	HADS— Depression	Fear of Cancer Recurrence Inventory (FCRI)	Insomnia
Fear of Cancer Recurrence inventory (FCRI)	r	0.76	0.64	-	0.585
	*p*	**<0.001**	**<0.001**	-	**<0.001**
Meaning	r	−0.36	−0.39	−0.23	−0.301
	*p*	**<0.001**	**<0.001**	**0.001**	**<0.001**
Peace	r	−0.64	−0.64	−0.54	−0.578
	*p*	**<0.001**	**<0.001**	**<0.001**	**<0.001**
Faith	r	−0.19	−0.28	−0.13	−0.152
	*p*	**0.005**	**<0.001**	0.053	0.025
Spirituality	r	−0.47	−0.54	−0.36	−0.411
	*p*	**<0.001**	**<0.001**	**<0.001**	**<0.001**
Insomnia	r	0.688	0.784	0.585	-
	*p*	**<0.001**	**<0.001**	**<0.001**	-

**Table 4:** Multiple regression results with depression, anxiety, and insomnia as dependent variables and fear of cancer recurrence as an independent variable, adjusted for demographics and other sample characteristics.

	Depression				Anxiety				Insomnia			
FCRI	β (SE) ^a^	t	*p*	95%Cl	β (SE) ^a^	t	*p*	95%Cl	β(SE) ^a^	t	*p*	95%Cl
	0.616 (0.031)	11.321	<0.001	0.290–0.412	0.777 (0.028)	16.519	<0.001	0.410–0.522	0.488 (0.034)	8.540	<0.001	0.223–0.357
	*F(12,203) = 16.950 R^2^ 47.1%*				*F(12,203) = 28.470 R^2^ 60.5%*				*F(12,203) = 13.795 R^2^ 41.2%*			

^a^ Regression coefficient (standard error) adjusted for demographics and other sample characteristics.

**Table 5:** Multiple regression results with fear of cancer recurrence and spirituality dimensions as independent variables, and depression, anxiety, and insomnia as dependent variables, adjusted for demographics and other sample characteristics.

					95% CI	
		β (SΕ)	t	*p*	Lower	Upper
Fear of cancer recurrence	Meaning	0.053 (0.225)	0.730	0.466	−0.279	0.607
	Peace	−0.573 (0.203)	−7.307	**<0.001**	−1.883	−1.083
	Faith	0.054 (0.164)	0.752	0.453	−0.200	0.447
*F(14,201) = 9.731 R^2^ 36.2%*						
Insomnia	Meaning	0.018 (0.127)	0.258	0.797	−0.218	0.284
	Peace	−0.513 (0.115)	−6.879	**<0.001**	−1.017	−0.564
	Faith	0.034 (0.093)	0.488	0.626	−0.138	0.229
*F(14,201) = 12.216 R^2^ 42.2%*						
Anxiety	Meaning	−0.075 (0.126)	−1.114	0.267	−0.389	0.108
	Peace	−0.598 (0.114)	−8.173	**<0.001**	−1.154	−0.705
	Faith	0.026 (0.092)	0.385	0.701	−0.146	0.217
*F(14,201) = 13.260 R^2^ 44.4%*						
Depression	Meaning	−0.132 (0.110)	−2.115	**0.036**	−0.451	−0.016
	Peace	−0.448 (0.100)	−6.629	**<0.001**	−0.858	−0.465
	Faith	−0.154 (0.081)	−2.480	**0.014**	−0.359	−0.041
*F(14,201) = 18.040 R^2^ 52.6%*						

β Regression coefficient (standard error) adjusted for demographics and other sample characteristics.

## Data Availability

Data are available from the corresponding author upon reasonable request.

## Public Involvement Statement

There was no public involvement in any aspect of this research.

## Guidelines and Standards Statement

This manuscript adheres to STROBE guidelines for observational studies.

## References

[1] Yang Y., Wen Y., Bedi C., Humphris G. (2017). The relationship between cancer patient’s fear of recurrence and chemotherapy: A systematic review and meta-analysis. J. Psychosom. Res..

[2] Simard S., Savard J., Ivers H. (2010). Fear of cancer recurrence: Specific profiles and nature of intrusive thoughts. J. Cancer Surviv..

[3] Lebel S., Ozakinci G., Humphris G., Mutsaers B., Thewes B., Prins J., Dinkel A., Butow P. (2016). From normal response to clinical problem: Definition and clinical features of fear of cancer recurrence. Support. Care Cancer.

[4] Lasry J.C., Margolese R.G. (1992). Fear of recurrence, breast-conserving surgery, and the trade-off hypothesis. Cancer.

[5] Lebel S., Tomei C., Feldstain A., Beattie S., McCallum M. (2013). Does fear of cancer recurrence predict cancer survivors’ health care use?. Support. Care Cancer.

[6] Hart S.L., Latini D.M., Cowan J.E., Carroll P.R., CaPSURE Investigators (2008). Fear of recurrence, treatment satisfaction, and quality of life after radical prostatectomy for prostate cancer. Support. Care Cancer.

[7] Avis N.E., Smith K.W., McGraw S., Smith R.G., Petronis V.M., Carver C.S. (2005). Assessing quality of life in adult cancer survivors (QLACS). Qual. Life Res..

[8] Simard S., Thewes B., Humphris G., Dixon M., Hayden C., Mireskandari S., Ozakinci G. (2013). Fear of cancer recurrence in adult cancer survivors: A systematic review of quantitative studies. J. Cancer Surviv..

[9] Mirzaei F., Farshbaf-Khalili A., Nourizadeh R., Zamiri R.E. (2021). Quality of life and its predictors in Iranian women with breast cancer undergoing chemotherapy and radiotherapy. Indian J. Cancer.

[10] Shin J., Shin D.W., Lee J., Hwang J., Lee J.E., Cho B., Song Y.M. (2022). Exploring socio-demographic, physical, psychological, and quality of life-related factors related with fear of cancer recurrence in stomach cancer survivors: A cross-sectional study. BMC Cancer.

[11] Koral L., Cirak Y. (2021). The relationships between fear of cancer recurrence, spiritual well-being and psychological resilience in non-metastatic breast cancer survivors during the COVID-19 outbreak. Psychooncology.

[12] Hong S.J., Shin N.-M., Jung S. (2020). A predictive model of fear of cancer recurrence for patients undergoing chemotherapy. Support. Care Cancer.

[13] Simonelli L.E., Siegel S.D., Duffy N.M. (2016). Fear of cancer recurrence: A theoretical review and its relevance for clinical presentation and Management. Psychooncology.

[14] Mardani A., Farahani M.A., Khachian A., Vaismoradi M. (2023). Fear of Cancer Recurrence and Coping Strategies among Prostate Cancer Survivors: A Qualitative Study. Curr. Oncol..

[15] Martin C.M., Greene D., Harrell J.P., Mwendwa D.T., Williams C.D., Horton S., Cradle M., Hudson B.D., Taylor T.R. (2020). The impact of social constraints on insomnia among African-American breast cancer survivors: The mediating role of fear of recurrence. Psychooncology.

[16] Savard J., Ivers H. (2013). The evolution of fear of cancer recurrence during the cancer care trajectory and its relationship with cancer characteristics. J. Psychosom. Res..

[17] Petzel M.Q., Parker N.H., Valentine A.D., Simard S., Nogueras-Gonzalez G.M., Lee J.E., Pisters P.W., Vauthey J.N., Fleming J.B., Katz M.H. (2012). Fear of cancer recurrence after curative pancreatectomy: A cross-sectional study in survivors of pancreatic and periampullary tumors. Ann. Surg. Oncol..

[18] Thewes B., Butow P., Zachariae R., Christensen S., Simard S., Gotay C. (2012). Fear of cancer recurrence: A systematic literature review of self-report measures. Psychooncology.

[19] Simard S., Savard J. (2015). Screening and comorbidity of clinical levels of fear of cancer recurrence. J. Cancer Surviv..

[20] Bredle J.M., Salsman J.M., Debb S.M., Arnold B.J., Cella D. (2011). Spiritual well-being as a component of health-related quality of life: The functional assessment of chronic illness therapy—Spiritual well-being scale (FACIT-Sp). Religions.

[21] Fradelos E.C., Tzavella F., Koukia E., Tsaras K., Papathanasiou I.V., Aroni A., Alikari V., Ralli M., Bredle J., Zyga S. (2016). The translation, validation and cultural adaptation of functional assessment of chronic illness therapy-spiritual well-being 12 (facit-sp12) scale in Greek language. Mater. Sociomed..

[22] Soldatos C.R., Dikeos D.G., Paparrigopoulos T.J. (2003). The diagnostic validity of the Athens Insomnia Scale. J. Psychosom. Res..

[23] Zigmond A.S., Snaith R.P. (1983). The hospital anxiety and depression scale. Acta Psychiatr. Scand..

[24] Michopoulos I., Douzenis A., Kalkavoura C., Christodoulou C., Michalopoulou P., Kalemi G., Fineti K., Patapis P., Protopapas K., Lykouras L. (2008). Hospital Anxiety and Depression Scale (HADS): Validation in a Greek general hospital sample. Ann. Gen. Psychiatry.

[25] Luigjes-Huizer Y.L., Tauber N.M., Humphris G., Kasparian N.A., Lam W.W.T., Lebel S., Simard S., Smith A.B., Zachariae R., Afiyanti Y. (2022). What is the prevalence of fear of cancer recurrence in cancer survivors and patients? A systematic review and individual participant data meta-analysis. Psychooncology.

[26] Crist J.V., Grunfeld E.A. (2013). Factors reported to influence fear of recurrence in cancer patients: A systematic review. Psychooncology.

[27] Niu L., Liang Y., Niu M. (2019). Factors influencing fear of cancer recurrence in patients with breast cancer: Evidence from a survey in Yancheng, China. J. Obstet. Gynaecol. Res..

[28] Kuswanto C.N., Sharp J., Stafford L., Schofield P. (2023). Fear of cancer recurrence as a pathway from fatigue to psychological distress in mothers who are breast cancer survivors. Stress Health.

[29] Quan L., Wang X., Lu W., Zhao X., Sun J., Sang Q. (2022). The relationship between fear of recurrence and depression in patients with cancer: The role of invasive rumination and catastrophizing. Front. Psychiatry.

[30] Pedersen A.F., Rossen P., Olesen F., von der Maase H., Vedsted P. (2012). Fear of recurrence and causal attributions in long-term survivors of testicular cancer. Psychooncology.

[31] Yang Y., Sun H., Luo X., Li W., Yang F., Xu W., Ding K., Zhou J., Liu W., Garg S. (2022). Network connectivity between fear of cancer recurrence, anxiety, and depression in breast cancer patients. J. Affect. Disord..

[32] Koch L., Bertram H., Eberle A., Holleczek B., Schmid-Höpfner S., Waldmann A., Zeissig S.R., Brenner H., Arndt V. (2014). Fear of recurrence in long-term breast cancer survivors-still an issue. Results on prevalence, determinants, and the association with quality of life and depression from the cancer survivorship-a multi-regional population-based study. Psychooncology.

[33] Lee-Jones C., Humphris G., Dixon R., Hatcher M.B. (1997). Fear of cancer recurrence—A literature re-view and proposed cognitive formulation to explain exacerbation of recurrence fears. Psychooncology.

[34] Hall D.L., Jimenez R.B., Perez G.K., Rabin J., Quain K., Yeh G.Y., Park E.R., Peppercorn J.M. (2019). Fear of Cancer Recurrence: A Model Examination of Physical Symptoms, Emotional Distress, and Health Behavior Change. J. Oncol. Pract..

[35] Perndorfer C., Soriano E.C., Siegel S.D., Spencer R.M.C., Otto A.K., Laurenceau J.P. (2022). Fear of cancer recurrence and sleep in couples coping with early-stage breast cancer. Ann. Behav. Med..

[36] Sinha A.K., Kumar S. (2014). Integrating spirituality into patient care: An essential element of modern healthcare system. Indian Heart J..

[37] Pargament K.I. (1997). The Psychology of Religion and Coping: Theory, Research, Practice.

[38] Carlson C.R., Bacaseta P.E., Simanton D.A. (1988). A controlled evaluation of devotional meditation and progressive relaxation. J. Psychol. Theol..

[39] Nelson C., Jacobson C.M., Weinberger M.I., Bhaskaran V., Rosenfeld B., Breitbart W., Roth A.J. (2009). The role of spirituality in the relationship between religiosity and depression in prostate cancer patients. Ann. Behav. Med..

[40] Fradelos E.C., Alikari V., Tsaras K., Papathanasiou I.V., Tzavella F., Papagiannis D., Zyga S. (2021). Assessment of psychological distress in end stage renal disease: Is it spirituality related?. Med. Pharm. Rep..

